# Use of social media for sexual health promotion: a scoping review

**DOI:** 10.3402/gha.v9.32193

**Published:** 2016-09-19

**Authors:** Elia Gabarron, Rolf Wynn

**Affiliations:** 1 Norwegian Centre for eHealth Research, University Hospital of North Norway, Tromsø, Norway; 2 Department of Clinical Medicine, Faculty of Health Sciences, The Arctic University of Norway, Tromsø, Norway; 3 Division of Mental Health and Addictions, University Hospital of North Norway, Tromsø, Norway

**Keywords:** social media, social networking sites, sexual health, health education, health promotion, sexually transmitted infections

## Abstract

**Background:**

In order to prevent sexually transmitted infections (STIs), the World Health Organization recommends educating people on sexual health. With more than 2 billion active users worldwide, online social media potentially represent powerful channels for health promotion, including sexual health.

**Objective:**

To review the scientific literature on the use of online social media for sexual health promotion.

**Design:**

A search was conducted of scientific and medical databases, and grey literature was also included. The selected publications were classified according to their study designs, sexual health promotion main subject, target audience age, and social media use.

**Results:**

Fifty-one publications were included; 4 publications presenting randomized intervention studies, 39 non-randomized intervention studies, and 8 observational studies. In 29 publications (56.9%), the main subject of the sexual health promotion was ‘general’ or to increase STI testing. Thirty publications (58.8%) specifically focused on youth or young people (aged 11–29 years). Fourteen publications that used social media either as unique channels for sexual health promotion interventions or as a tool supporting the sexual health promotion reported an effect on behavior (27%), and two of those studies found a reduction in the number of positive chlamydia and gonorrhea cases linked to social media intervention. Forty-four publications (86.3%) involved Facebook in some way.

**Conclusions:**

Although billions of people worldwide actively use social media, we identified only 51 publications on the use of social media for promoting sexual health. About a quarter of the publications have identified promising results, and the evidence for positive effects of social media interventions for promoting sexual health is increasing. There is a need for more studies that explicitly discuss their theoretical framework, and that have strong research designs, in order to further increase the evidence base of the field.

## Introduction

It is estimated that about a million people globally acquire a sexually transmitted infection (STI) every day, including many who are infected with the human immunodeficiency virus (HIV) (1, 2). Individuals who have STIs may experience acute or chronic symptoms. Women with STIs in particular are at risk for pelvic inflammatory disease, cervical cancer, infertility, ectopic pregnancy, and transmitting STIs to their children during birth (1, 2). In order to prevent STIs, minimize the effects among infected people, and to reduce the enormous burden that STIs represent for developed and developing countries, the World Health Organization (WHO) has published the *Global Strategy for the Prevention and Control of Sexually Transmitted Infections*, where the need for educating people on sexual health is emphasized (1, 2). In order to achieve this educational goal, the WHO points to the importance of choosing the communication channels that most effectively reach the target population (1, 2).

Online social media, such as Facebook or Twitter, have become extremely popular worldwide and might therefore be powerful channels for reaching many people. Since their launch, the adoption of these technologies has been steeply increasing, surpassing 2 billion active users worldwide in 2015. Facebook is the most used social media channel, with more than 1.5 billion users (3). Because social media are popular and frequently used by many people of various ages worldwide, there is potential for the media to be used for health promotion (4–7), including for potentially sensitive and stigmatizing subjects such as those related to sexual health (8–10). Previous reviews have addressed the use of computer-based technologies in general for sexual health promotion but not specifically the social media (11–14). And some reviews have analyzed the use of social media for health promotion in general, including sexual health (8, 10). We have identified prior reviews that addressed the use of online social media specifically for sexual health education or sexual health promotion. One was an early (2011) review of the literature that found that 71% of the earliest promotion activities used Facebook, 30% targeted young people, and 25% specifically addressed HIV (15). A more recent review focused specifically on the impact of social media interventions targeting adolescents and young adults. The study reported that online social media can increase knowledge regarding STI prevention in this population, but the evidence was weaker regarding the effects on behavior change (16). Another review studied the viability of social media as tools that health care professionals can provide to adolescents (17). The authors highlighted the need for further studies on how to use these technologies to educate adolescents about STIs (17).

The number of publications and programs describing social media as a means for promoting sexual health is rapidly increasing. There is a need for an updated review of the literature that is not restricted to youth or to peer-reviewed publications, specific uses of social media, or to specific types of outcomes or study designs, that examines the literature regarding the use of social media for STI prevention and health promotion. The objective of this scoping review is to describe the scientific literature on the use of online social media for sexual health education and sexual health promotion.

## Methods

To analyze the use of online social media for sexual health education or sexual health promotion, we used a systematic approach, drawing on Preferred Reporting Items for Systematic Reviews and Meta-Analyses (PRISMA) guidelines (18). A full electronic search strategy covered all the studies published until the end of October 2015 involving the terms ‘sexual health promotion’ or ‘sexual health education’ in combination with the following words: ‘social media’; ‘social networking’; ‘Facebook’; ‘Twitter’; ‘YouTube’; ‘Instagram’; and ‘Snapchat’. The search was performed in the following multidisciplinary databases: Embase, Pubmed (MeSH terms and text word), PsychINFO, Applied Social Sciences Index and Abstracts (ASSIA), ProQuest Health and Medical Complete, British Nursing Index, Computer and Information System Abstracts, and MEDLINE (Ovid).

To capture grey literature, additional publications, conference proceedings, and research reports were searched in additional databases (e.g. African Journals Online [AJOL], COS Conference Papers Index, Directory of Open Access Journals [DOAJ], and ClinicalTrials.gov). Abstracts presented at the following conferences and published in the journals *Sexually Transmitted Diseases*, *Sexually Transmitted Infections*, and *International Journal of STD & AIDS* were also scanned: 4th joint BASHH-ASTDA meeting, 2012; BASHH 2013; STI & HIV World Congress 2013; 2014 STD Prevention Conference; Infection Prevention 2014; BASHH 2015; STI & HIV World Congress 2015; 29th European Conference on Sexually Transmitted Infections, 2015.

We also searched manually for program evaluation reports referring to the use of social media for sexual health promotion or sexual health education and available on websites of the following non-governmental organizations (NGOs): International Planned Parenthood Federation (IPPF); Population Council; WHO; United Nations Population Fund (UNFPA); Youth Leading the HIV & Hep C Movement (YouthCO); FHI360; Phoenix PLUS and menZDRAV Foundation; and The Initiative for Equal Rights (TIER). The full search strategy is summarized in the Supplementary file.

Publications were included in the review if they: 1) were empirical studies reporting results, 2) described studies that used social media as a tool for sexual health promotion or education, and 3) were written in English. Papers that did not meet all three criteria were excluded.

Following the search, duplicates were removed. Thereafter, all the titles and abstracts were examined by one reviewer (EG) to determine if the papers met the inclusion criteria; doubts regarding their inclusion/exclusion were discussed and agreed with a second reviewer (RW). Subsequently, full-text articles of the selected studies were retrieved and rigorously examined to sort out any remaining papers that did not meet the criteria. The articles selected for full review were classified according to their study designs (i.e. randomized or, non-randomized intervention studies or observational studies); main sexual health promotion subject (incurable STI, HIV; curable STIs, i.e. chlamydia/syphilis/gonorrhea/human papillomavirus (HPV); or STI prevention or sexual health in general); target audience age (specifically youths or young people; adults; or unspecified/general); sexual preference (straight/unspecified/all; men who have sex with men [MSM]; lesbian, gay, bisexual, and transgender [LGBT]); and social media use (as a unique channel for the promotion or as a tool supporting sexual health promotion). Data were extracted by one reviewer (EG) and verified by a second reviewer (RW).

## Results

### Sample

**Fig. 1:**
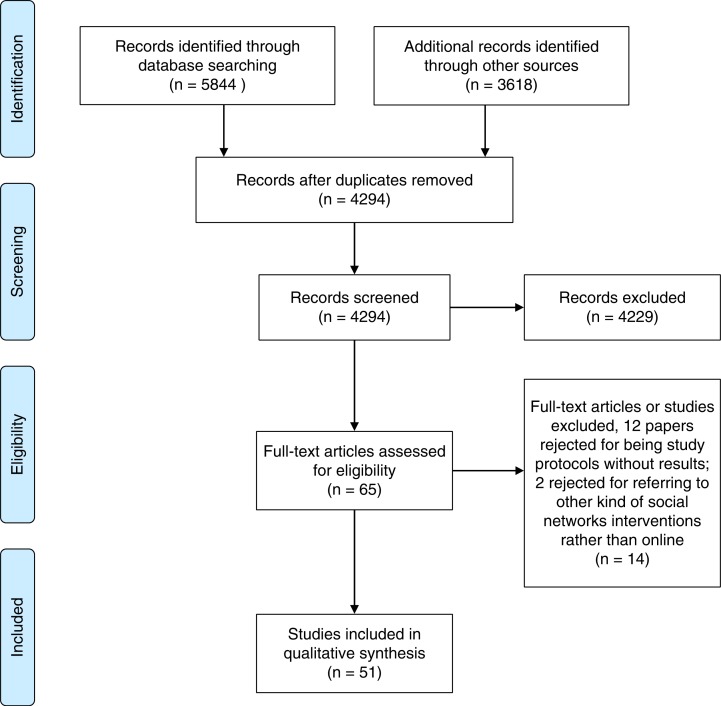
Flowchart of the selection procedure.

A total of 9,462 publications were identified; the search strategy and its results are summarized in the Supplementary file. Fifty-one of these publications met the inclusion criteria (19–69) (see Fig. 1). Forty-one of these 51 publications corresponded to unique studies; in addition, three publications belonged to Project HOPE (26, 40, 43); three were part of the Get Yourself Tested campaign (19, 37, 50); two papers referred to the FaceSpace Project (23, 33); and two studies reported on an HIV self-test campaign on Grindr. All the included studies were carried out between 2008 and 2015.

### Designs of included publications

Among the 51 included publications, 4 reported on randomized intervention studies, 39 reported on non-randomized interventions, and 8 reported on observational studies. A summary of the publications included in this review is presented in Table 1.

**Table 1:** Summary of publications included in the review (*n*=51)

Reference	Study design	Sexual health subject	Target audience	Social media use	Media	Outcomes	Country
Bull et al., 2012 (22)	Randomized (cluster RCT)	Sexual health promotion/STI prevention (general)	1,578 participants (16–25 years old)	Social media is the only channel for promotion.	Facebook	75% completed at least one study follow-up. At 2 months, the study reports significantly more condom use (68% in the intervention group vs. 56% in control group, *p*=0.04) and more sex acts protected with condoms (intervention 63% vs. control 57%, *p*=0.03). No effects were seen at 6 months.	US
Young et al., 2013 (26)	Randomized	Incurable STI (HIV)	16 peer health educators, African American and Latino MSM from Los Angeles	Social media is the only channel for promotion.	Facebook, MySpace	After the training, the peer leaders were significantly (*p*=0.03) more comfortable using social media to discuss sexual health topics. There were no significant differences pre- and post-training on other comfort or knowledge measures because almost all peer leaders were already using social media at baseline.	US
Young et al., 2014 (40)	Randomized	Incurable STI (HIV)	Racial/ethnic minority MSM, 18 years of age or older	Social media is the only channel for promotion.	Facebook	There was a significant relation between increase in network ties and use of social media to discuss sexual behaviors and partners and a positive trending relationship (*p*<0.1) among increase in network ties and likelihood of testing for HIV, follow-up for test results, and participation in group discussions. There were no significant differences among the control groups.	US
Patel et al., 2014 (38)	Randomized (cluster RCT)	Curable STIs (chlamydia/syphilis/gonorrhea/HPV)	365 women (19–26 years old)	As resource/tool supporting sexual health promotion	Facebook	Reminder system did not increase completion rates (intervention 17.2% vs. control 18.9%, *p*=0.88). Participants that completed the series on time were more likely to be older (OR=1.15, 95% CI 1.01–1.31), to have completed a 4-year college degree or more (age-adjusted OR=2.51, 95% CI 1.29–4.90), and to report three or more life-time sexual partners (age-adjusted OR=3.45, 95% CI 1.20–9.92).	US
Population Council, 2010 (60)	Non-randomized intervention	Incurable STI (HIV)	MSM (age range not specified)	As resource/tool supporting sexual health promotion	Not specified	The program reached 21,000 high-risk men with HIV prevention messages and tested about 7,000 men for HIV.	Nigeria
Friedman et al., 2011 (19)	Non-randomized intervention	Sexual health promotion/STI prevention (general)	Young people (15–25 years)	As resource/tool supporting sexual health promotion	YouTube (and tracked with Twitter)	Campaign potentially reached 4,000 health centers nationwide. Campaign reported 71% increase in patients presenting for STI testing.	US
Gold et al., 2012 (23)	Non-randomized intervention	Sexual health promotion/STI prevention (general)	Two groups: young people (16–29 years old), and men who have sex with men	Social media is the only channel for promotion.	Facebook, Twitter, Flickr, YouTube	Five Facebook pages had 900 fans; 31 YouTube videos had 5,300 views.	Australia
FHI360, 2013 (66)	Non-randomized intervention	Incurable STI (HIV)	Men who have sex with men (age range not specified)	Social media is the only channel for promotion.	Facebook, Whatsapp, other social media platforms	More than 15,000 MSM were reached through social media, most of them people that peer educators would not usually reach	Ghana
TIER, 2013 (67)	Non-randomized intervention	Incurable STI (HIV)	Men who have sex with men (age range not specified)	As resource/tool supporting sexual health promotion	Not specified	More than 5,000 MSM living with HIV have received information and services; 73% of MSM reached through the program report correct and consistent use of condoms (vs. 43% at the inception of the program).	Nigeria
Phoenix PLUS and menZDRAV Foundation, 2013 (68)	Non-randomized intervention	Incurable STI (HIV)	MSM living with HIV (18–25 years old)	As resource/tool supporting sexual health promotion	Facebook, Vkontakte	Around 3,000 MSM living with HIV received information. Counselors provided 1,900 phone consultations and 1,350 online consultations. The website received about 15,000 visitors.	Russia
Mustanski, 2013 (32)	Non-randomized intervention	Sexual health promotion/STI prevention (general)	LGBT youth in same-sex relationship (16–20 years old)	As resource/tool supporting sexual health promotion	Facebook	*N*=202 LGBT youth completed the intervention, with a mean completion time of 106 min. No effect sizes reported.	US
Gabarron et al., 2013 (28)	Non-randomized intervention	Sexual health promotion/STI prevention (general)	Youth, web app users from northern Norway	As resource/tool supporting sexual health promotion	Facebook, Twitter	70% of web app uses were returning visitors, and they spent an average of 7 min on the site.	Norway
Pedrana et al., 2013 (34)	Non-randomized intervention	Sexual health promotion/STI prevention (general)	Young people aged 16–29 years and gay men	Social media is the only channel for promotion.	Facebook, YouTube	Almost 3,000 Facebook fans and more than 30,000 YouTube video views.	Australia
Nguyen et al., 2013 (33)	Non-randomized intervention	Sexual health promotion/STI prevention (general)	Young people (16–29 years)	Social media is the only channel for promotion.	Facebook, YouTube, MySpace, Twitter, Flickr	The study had 900 fans. The most successful way of increasing audience reach were via Facebook advertisements and tagging photos of young people attending music festivals on Facebook pages.	Australia
Fisser, 2013 (55)	Non-randomized intervention	Sexual health promotion/STI prevention (general)	Teenagers (no age range specified)	Social media is the only channel for promotion.	General social media use	Campaign reached 91% of targeted population. After the campaign, 74% of youths reported using condoms (vs. 56% before campaign).	Netherlands
IPPF, 2013 (62)	Non-randomized intervention	Sexual health promotion/STI prevention (general)	General population (age range not specified)	Social media is the only channel for promotion.	YouTube	The film had more than 75,000 views.	Ireland
Prior et al., 2014 (46)	Non-randomized intervention	Sexual health promotion/STI prevention (general)	Youth, aged 13–24 years	As resource/tool supporting sexual health promotion	Facebook	Knowledge about where to get free condoms increased from 58 to 70%. No differences were found in the percentage of condom use at baseline and after the campaign.	US
Anderson and Samplin-Salgado, 2014 (50)	Non-randomized intervention	Sexual health promotion/STI prevention (general)	Youth people (age ranges not specified)	As resource/tool supporting sexual health promotion	Facebook	A random sample survey showed that 23% of youths had heard about the prevention program.	US
Klingler, 2014 (48)	Non-randomized intervention	Sexual health promotion/STI prevention (general)	Young people (18–29 years old)	As resource/tool supporting sexual health promotion	Facebook	The website received about 50,000 unique visitors monthly.	US
Friedman et al., 2014 (37)	Non-randomized intervention	Sexual health promotion/STI prevention (general)	Sexually active young women (15–25 years old) and their partners	As resource/tool supporting sexual health promotion	Facebook, Twitter	There was an increase in the number of people tested for chlamydia during the campaign (increase ranged from 0.5 to 128%).	US
UNFPA, 2014 (59)	Non-randomized intervention	Sexual health promotion/STI prevention (general)	Young people (age range not specified)	As resource/tool supporting sexual health promotion	Not specified	The Y-PEER program reached thousands of young people around the Arab States, providing training and educational opportunities to learn about sexual health.	Arab States (22 countries in Middle East and North Africa)
IPPF, 2014 (61)	Non-randomized intervention	Sexual health promotion/STI prevention (general)	Women and girls (age range not specified)	As resource/tool supporting sexual health promotion	Not specified	More than 2,500 women and girls attended 144 tea parties, where the sexual health education took place.	Pakistan
YouthCO, 2014 (65)	Non-randomized intervention	Sexual health promotion/STI prevention (general)	Young MSM (age range not specified)	As resource/tool supporting sexual health promotion	Facebook, Twitter, Instagram	The project reached hundreds of participants. Each event was attended by about 10–20 men.	Canada
IPPF, 2015 (63)	Non-randomized intervention	Sexual health promotion/STI prevention (general)	Young people (age range not specified)	As resource/tool supporting sexual health promotion	Not specified	Materials were distributed to more than 2,000 people. Young people's understanding of issues relating to sexual and reproductive health and rights increased. During the project, the youth center assisted 415 women with pregnancy-related needs, 249 of them were referred to abortion clinics.	Spain
IPPF, 2015 (64)	Non-randomized intervention	Sexual health promotion/STI prevention (general)	Young people (age range not specified)	Social media is the only channel for promotion.	Not specified	Materials and messages reached more than half a million social media users.	Macedonia
IPPF, 2015 (69)	Non-randomized intervention	Sexual health promotion/STI prevention (general)	Young people (age range not specified)	As resource/tool supporting sexual health promotion	Facebook, Twitter, Whatsapp, YouTube	The project reached more than 2,000 people.	Ghana
Dowshen et al., 2015 (42)	Non-randomized intervention	Sexual health promotion/STI prevention (general)	Youth (13–17 years old)	As resource/tool supporting sexual health promotion	Facebook, Twitter, Instagram, YouTube	70% of surveyed users said they intended to test in 6 months.	US
Chu et al., 2015 (41)	Non-randomized intervention	Sexual health promotion/STI prevention (general)	788 school students (12–16 years of age)	As resource/tool supporting sexual health promotion	Facebook	The game was well-received by adolescents. Student responses indicated a link between gameplay and potential for behavior change.	Hong Kong
Veale et al., 2015 (45)	Non-randomized intervention	Sexual health promotion/STI prevention (general)	60 Facebook and 40 Twitter profiles promoting sexual health (unspecified age)	Social media is the only channel for promotion.	Facebook, Twitter	The top 10 ranked profiles made regular posts/tweets (46 posts or 124 tweets/month); individualized interaction with users; encouraged interaction and conversation by posting questions and highlighting celebrity involvement.	Australia
Fuller and Carter, 2015 (58)	Non-randomized intervention	Sexual health promotion/STI prevention (general)	Young people from Western Australia (no age range defined)	As resource/tool supporting sexual health promotion	Facebook	Young participants reported increases in their knowledge of sexual health, in blood-borne virus issues, and feeling more confident about related issues.	Australia
Staub et al., 2015 (54)	Non-randomized intervention	Sexual health promotion/STI prevention (general)	Sexually active population (age range not specified)	As resource/tool supporting sexual health promotion	General social media use	The website was visited by 270,000 people, and the spot was seen more than 1 million times. This represented about 18% of the targeted population.	Switzerland
Day and Hughes, 2012 (56)	Non-randomized intervention	Incurable STI (HIV)	Men aged 18–50 years, single or in a relationship living within 50 miles from London	Social media is the only channel for promotion.	Facebook	The advertising campaign reached 8.5% of the targeted population.	UK
Hildebrand et al., 2013 (29)	Non-randomized intervention	Incurable STI (HIV)	3,497 young people (15–29 years old) from 79 countries participated in the online forums; 1,605 participants in the offline forums	As resource/tool supporting sexual health promotion	General social media use	The study enabled thousands of young people to engage in discussions on issues of HIV and sexuality.	79 countries
Ko et al., 2013 (30)	Non-randomized intervention	Incurable STI (HIV)	2,074 men (older than 18)	Social media is the only channel for promotion.	Facebook	There were 432 articles posted by 369 opinion leaders. At 6 months, MSM visiting the intervention website reported being more likely to receive HIV-related information (25.5% vs. 10.5%, *p*<0.001), to have HIV tests (43.9% vs. 22.3%, *p*<0.001), and to use condoms (34.2% vs. 26.2%, *p*<0.005).	Taiwan
Menacho et al., 2015 (43)	Non-randomized intervention	Incurable STI (HIV)	34 MSM peer leaders (19–45 years old)	Social media is the only channel for promotion.	Facebook	Peer leaders completed training sessions consisting of secret Facebook groups, with 28–32 participants each and 5–6 peer leaders in each group, for 12 weeks.	Peru
West and Daniels, 2015 (52)	Non-randomized intervention	Incurable STI (HIV)	MSM and use Grindr (no age range specified)	Social media is the only channel for promotion.	Grindr	55 users booked an appointment, and 34 attended. The appointment service proved to be effective in attracting new service users who were less likely to have utilized STI testing services.	UK
Huang et al., 2015 (51)	Non-randomized intervention	Incurable STI (HIV)	African American and Latino men, older than 18 years	Social media is the only channel for promotion.	Grindr	The HIV self-test was utilized by 455 users. Survey responses were obtained from 112 participants, four of them reported as HIV positive.	US
Huang et al., 2015 (53)	Non-randomized intervention	Incurable STI (HIV)	African American and Latino men, older than 18 years	Social media is the only channel for promotion.	Grindr	HIV self-testing promotion on Grindr resulted in 667 HIV self-test requests.	US
Jones et al., 2012 (24)	Non-randomized intervention	Curable STIs (chlamydia/syphilis/gonorrhea/HPV)	15–24 years old	Social media is the only channel for promotion.	Facebook	There was a 23% self-reported increase in condom utilization and a 54% reduction in positive chlamydia cases among 15–17 year olds.	US
Coughlan et al., 2014 (36)	Non-randomized intervention	Curable STIs (chlamydia/syphilis/gonorrhea/HPV)	Adult population 19–48 years old	Social media is the only channel for promotion.	Facebook	High incidence of social media use for meeting contacts among the syphilis diagnosed cases.	New Zealand
Syred et al., 2014 (39)	Non-randomized intervention	Curable STIs (chlamydia/syphilis/gonorrhea/HPV)	Moderators and participants from a sexual health promotion site on Facebook targeting 15–24 years old	Social media is the only channel for promotion.	Facebook	The health promotion site provided a space for single user post but not for self-sustaining conversation.	UK
Gourley, 2014 (47)	Non-randomized intervention	Curable STIs (chlamydia/syphilis/gonorrhea/HPV)	MSM (age range not specified)	As resource/tool supporting sexual health promotion	General social media use	The number of syphilis tests increased from 719 to 879 during the campaign and the number of syphilis diagnoses increased from 23 to 41 (an increase of 78%).	US
Smith et al., 2014 (49)	Non-randomized intervention	Curable STIs (chlamydia/syphilis/gonorrhea/HPV)	Population from Androscoggin County (age range not defined)	As resource/tool supporting sexual health promotion	General social media use	A 51% decrease in gonorrhea cases was reported in a 9-month campaign (from 143 cases down to 69).	US
Selkie et al., 2011 (21)	Observational	Sexual health promotion/STI prevention (general)	29 adolescents (14–19 years old)	As resource/tool supporting sexual health promotion	General social media use	Adolescents were enthusiastic regarding technology for sexual health education. Adolescents showed preference for sexual health education resources that were accessible, trustworthy, and offered in a nonthreatening way.	US
Hedge et al., 2011 (20)	Observational	Sexual health promotion/STI prevention (general)	78 patients from sexual health services (15–25 years old)	Social media is the only channel for promotion.	Facebook, MySpace, Bebo, High5	Questionnaire to service users; 81% said they would use the group pages for sexual health information.	UK
Vyas et al., 2012 (25)	Observational	Sexual health promotion/STI prevention (general)	428 Latino adolescents from 12 high schools	As resource/tool supporting sexual health promotion	General social media use, including Facebook, MySpace, Twitter, YouTube, and others	SMS and social media were pervasive among Latino youth. Facebook was related to positive concepts (youth have access to it, and they check it every day; it is an easy communication channel and has open access) and to negative concepts (potential risk for cyberbullying or inappropriate content).	US
Bull et al., 2013 (27)	Observational	Sexual health promotion/STI prevention (general)	7,500 pupils (11–16 years old)	As resource/tool supporting sexual health promotion	Twitter	Opportunities to dispel multiple myths—many of which were perpetrated via uncensored social media.	UK
Wohfeiler et al., 2013 (35)	Observational	Sexual health promotion/STI prevention (general)	Dating website users, website owners, health department HIV/STD directors (unspecified age)	As resource/tool supporting sexual health promotion	General social media use	The majority of stakeholders in the three groups would not agree with interventions including links to social media (such as Facebook).	US
Nasution, 2013 (57)	Observational	Incurable STI (HIV)	MSM and transgender from four countries	Social media is the only channel for promotion.	Own created social media ‘PlaySafe’ and ‘Peer Support’	Users reported concerns with the registration and the need to identify themselves as MSM or transgender. Users suggested using local images, including links to other websites, and providing feedback on the online post-tests.	Indonesia, Malaysia, Philippines, and Timor-Leste
Ramallo et al., 2015 (44)	Observational	Incurable STI (HIV)	MSM (> 18 years old)	Social media is the only channel for promotion.	General social media use	The main obstacle to effective HIV prevention campaigns in social networks was stigmatization based on homosexuality and HIV status.	US
McDaid et al., 2013 (31)	Observational	Curable STIs (chlamydia/syphilis/gonorrhea/HPV)	60 heterosexual young men (aged 16–24 years)	As resource/tool supporting sexual health promotion	General social media use	Participants reacted favorably to an online approach for accessing postal chlamydia tests.	UK

HIV, human immunodeficiency virus; HPV, human papillomavirus; LGBT, lesbian, gay, bisexual, and transgender; MSM, men who have sex with men; STI, sexually transmitted infection.

#### Randomized studies

Only four of the included papers had a design that involved a randomization procedure (22, 26, 38, 40). Two of these publications, belonging to the Project HOPE study, described an intervention in which participants were randomized to receive either peer-delivered HIV-related information or general health information through Facebook for 12 weeks, framed within a social network intervention and, specifically, a peer-delivered intervention (26, 40). The study reported that peer leaders felt more comfortable discussing sexual health on social media (93.3% and 100% vs. 68.8% and 93.8%, *p*<0.05) and also found a positive association between participation in the group and the likelihood of HIV testing (26, 40). Another study randomized the participants to an intervention through a Facebook page on youth health information or to a News page on Facebook for 2 months (22). The study found a higher tendency to use condoms in the intervention group at 2 months (intervention 68% versus control 56%, *p*<0.04) and more frequent protection in sex acts (intervention 63% versus control 57%, *p*<0.03) but no lasting effect at 6 months (*p*=0.86) (22). Another study randomized health centers to offer a reminder service to increase HPV vaccination completion (including messages sent through Facebook) or to schedule routine follow-up (38). The intervention group did not increase vaccine completion rates (38). All the randomized studies were carried out in the US.

It is difficult to compare the effects of these randomized studies because they measure outcomes as different as feeling comfortable discussing sexual health on social media and rates of HIV testing (26, 40), condom use (22), and HPV vaccination completion rates (38). Two of the three studies did report some type of positive statistically significant outcome from social media interventions.

#### Non-randomized intervention studies

Thirty-nine of the selected publications had a non-randomized design. Eighteen of them only showed data regarding project reach and engagement (number of users, time spent, etc.) (23, 28, 30, 43, 48, 54, 56, 59–69), while another 10 publications additionally showed data on STI incidence and testing (19, 24, 36, 37, 47, 49, 51–53, 55). The designs in 12 of the publications combined quantitative data on technology use and also questionnaires or opinions of project users regarding their satisfaction, knowledge, engagement, or behavior change (intention to test) (29, 30, 33, 34, 39, 41, 42, 46, 50, 58, 63, 67). The remaining study had an observational approach and analyzed the strategies for successful user engagement in some Facebook and Twitter profiles undertaking sexual health promotion (45).

Regarding the effect of these non-randomized interventions, two studies reported that chlamydia and gonorrhea infections were reduced by 54 and 51%, respectively, after the intervention (24, 49), while the number of syphilis cases was increased by 78% in another study as a result of an increased number of STIs tests (47). Three studies reported increases in the number of patients presenting for STI testing by 71% (19), 122% (47), and up to 128% (37); and one study found a significant increase in intention to test from 22.3 to 43.9% (30). As a result of the intervention, 34 and 249 program users, respectively, attended health services (52, 63) and 667 asked for a self-test (53).

Some non-randomized intervention studies also reported an increase in condom use (74% vs. 56%) (55) and (73% vs. 43%) (67); a 23% self-reported increase in condom utilization (24); or a significantly increased intention to use condoms (34.2% vs. 26.2%) (30).

Only five of these non-randomized intervention studies or programs referred to the theoretical models they used as a framework for behavior change intervention (24, 30, 41, 58, 65). The chosen approaches were Kelly's popular opinion leader model; game-based learning with participatory approach; Pender's health promotion model; peer education models; and a community engagement model. Two projects referring to the Kelly's popular opinion leader model and Pender's health promotion model, respectively, reported positive results regarding an increase in intention to test (43.9% vs. 22.3%) and in intention to use condoms (34.2% vs. 26.2%) (30); 23% self-reported an increase in condom utilization, and 54% reported a reduction in positive chlamydia cases among 15–17 years olds (24).

#### Observational studies

Eight studies were observational—where the subjects participated in focus groups or answered questionnaires or surveys (20, 21, 25, 27, 31, 35, 44, 57). In all the studies with young people, social media were reported to be pervasive, and the study participants reacted positively to using new technologies for sexual health promotion or education (20, 21, 25, 27, 31). In the observational studies with adults, the importance of considering privacy, stigma, and social norms was emphasized (44, 57), and in this sense, links to social media profiles were not considered to be appealing (35). No theoretical framework was reported in any of the observational studies included in this review.

### Sexual health promotion main subject

In 29 of the 51 publications (56.9%), the main subject of the sexual health promotion was ‘general’ or to increase STI testing. Fifteen publications (29.4%) focused specifically on the incurable STI, HIV (26, 29, 30, 40, 43, 44, 51–53, 56, 57, 60, 66–68), and seven publications (13.7%) addressed curable STIs such as chlamydia, syphilis, gonorrhea, or HPV (24, 31, 36, 38, 39, 47, 49).

### Target audience age

Among the 51 included publications, 30 (58.8%) specifically focused on sexual health education for youth or young people (11–29 years), while 11 publications (21.6%) explicitly targeted adults (including young adults and middle-aged and older adults). The 10 remaining publications (19.6%) were not directed to any specific age group (Table 2).

**Table 2:** Target age groups of the included publications (*n*=51)

	Youth/young^a^ (11–29)	Adults^b^ (>18)	Unspecified or general
Study type			
Randomized study	2	2	0
Non-randomized intervention study	23	8	8
Observational study	5	1	2
Sexual preference			
Straight/unspecified/all	25	0	5
MSM/LGBT	5	11	5
Sexual health promotion main subject			
Incurable STI (HIV)	2	10	3
Curable STIs (chlamydia/syphilis/gonorrhea/HPV)	4	1	2
Sexual health promotion/STI prevention (general)	24	0	5
Social media use			
As only strategy to promote sexual health	9	10	4
As resource/tool supporting a sexual health promotion (websites, games, on-air components, etc.)	21	1	6
Total	30	11	10

a Youth/young adult group refers to populations aged 11–29 years

b adults (>18) group refers to young adults and middle-aged and older adults. HIV, human immunodeficiency virus; HPV, human papillomavirus; LGBT, lesbian, gay, bisexual, and transgender; MSM, men who have sex with men; STI, sexually transmitted infection.

### Use of social media

In 23 of the included studies (45%), social media was the core or the unique channel used for sexual health promotion. These studies involved Facebook, either used separately (22, 24, 30, 36, 39, 40, 43, 56) or in addition to other social media channels (such as Twitter, Flickr, YouTube, etc.) (20, 23, 26, 33, 34, 44, 45, 55, 64, 66). Three studies used the geosocial networking app Grindr (51–53); one was carried out on YouTube (62); and one publication referred to two purpose-developed online social networks (57). Eight publications that used social media as unique channels for sexual health promotion interventions reported an effect on behavior (three randomized trials and five non-randomized interventions). The reported effects were: increased condom use (74% vs. 56% before the intervention; and 68% in the intervention group versus 56% in the control group, *p*<0.05), 23% self-reported an increase in condom use or intention to use condoms (34.2% vs. 26.2%); 34 social media users utilized health services; 667 users requested an HIV self-test and indicated an increased intention to test (43.9% vs. 22.3%); users were more comfortable using social media to discuss sexual health topics (*p*<0.05) (22, 24, 26, 30, 40, 52, 53, 55). One of these publications also found a 54% reduction in the number of positive chlamydia cases linked to social media intervention in a specific age group (24).

In 28 of the publications (55%), social media was considered as a resource supporting another sexual health promotion channel. Twenty of these 28 publications considered more than one social media channel as a supporting resource for the promotion (i.e. Twitter, Instagram, YouTube, Whatsapp, Vkontakte) or did not specify a unique resource (19, 21, 25, 28, 29, 31, 35, 37, 42, 47, 49, 54, 59–61, 63, 65, 67–69). Only one of these studies did not refer specifically to Facebook. In this latter study, the sexual health promotion was carried out on YouTube, and the comments on the study were tracked though Twitter (19). When a unique social media channel to support the promotion was chosen, Facebook was the medium of choice in seven publications (32, 38, 41, 46, 48, 50, 58) and Twitter in one study (27). Six publications using online social media as a tool supporting sexual health promotion reported an effect on behavior, all of them non-randomized interventions. The effects were 73% self-reporting condom use (versus 43% before the intervention) (67); 249 new users utilizing health services (63); and an increase in STI testing by 71 to 128% (19, 37, 47). Two publications reported a reduction in the number of cases of gonorrhea by 51% and also a 78% increase in syphilis cases as a result of the increase in syphilis tests (47, 49).

## Discussion

### Overview

The use of the Internet for health purposes has been increasing for a long time (70, 71), and many health services around the world now offer Internet-based services (16, 72). The development of online social media is more recent, but these media have become very popular (3), offering a strong potential for health-related use and also within the field of sexual health (15). The present review shows that although online social media have been used in the sexual health promotion research field, the number of scientific studies is still relatively modest; we were able to identify 51 publications that fit the inclusion criteria.

The use of social media for sexual health promotion is a rapidly emerging field. Although some reviews have previously been published dealing with the topic (10–17), these are either several years old, or they only report on selected target groups (i.e. youth, high-risk men, etc.). The four RCTs (Randomized Controlled Trials) reported on in the present review are relatively new and therefore were not included in several of the prior reviews. Our updated review includes recent publications covering all target groups. It also covers relevant sexual health promotion projects that have been carried out by NGOs, many of which have not been included in previous review papers.

### There is a need to include randomization procedures and to discuss theoretical frameworks

A main finding in this review was that 14 of the 51 publications (27%) reported a behavior change effect regarding sexual health that was linked to social media. However, only three of these publications were randomized studies (22, 26, 40), that is, studies with a design that allowed for controlled measurements of the effects of the interventions. Two of these quality studies (three publications) reported important results linked to the use of social media with regard to users feeling comfortable with sexual health topics on these channels (26, 40), a short-term increase in condom use, and increased HIV testing rates (22). Another randomized trial did not find any benefit in sending reminders to complete a HPV vaccination schedule with the use of several channels, including social media (38). Although the four studies had a strong RCT design, they all reported on various limitations that might have impacted the findings, including a reliance on self-reporting (17), small sample sizes (21, 35), and low completion rates (33). The different outcome measures used in the randomized studies make it difficult to compare the outcomes of the different social media interventions in these studies. However, we find it promising that two of the three studies did report positive statistically significant differences between the intervention and control groups.

Only 4 of the 14 studies reporting behavior effects have been contextualized within a theoretical framework or model or have referred explicitly to a model. More studies that systematically evaluate interventions with a randomized control condition and that refer to a theoretical framework clearly are needed to increase the evidence and move the field forward.

A further 39 studies were non-randomized intervention studies or programs, a design that allowed for the testing out of various interventions, but where it would be somewhat more difficult to make strong claims about the effects of the interventions themselves (because there was no randomization). The non-randomized intervention studies seemed to have had positive results in terms of increases in rates of STI testing, higher condom use, and intention to test, better sexual health knowledge, and a potential to change behavior linked to the intervention (19, 24, 30, 37, 47, 52, 53, 55, 63, 67), and even a change in curable STIs rates (24, 47, 49). Many of the studies reported that large numbers of people had visited educational sites or utilized the online interventions (23, 28, 29, 32–34, 39, 43, 45, 48, 50, 54, 56, 59–69).

Eight studies were observational and, although these designs may provide important insights—for instance, relating to willingness to use or obstacles to use online social media for sexual health (20, 21, 25, 27, 31, 35, 44, 57), they do not allow for strong claims about the effects of social media on STI prevention and sexual health promotion. This means that, although there have been some studies examining the effects of the use of social media in this field, there still is a lack of studies with a more rigorous design allowing for stronger claims about the effects of such interventions.

It must be highlighted that only seven publications referred to some type of theoretical framework or model upon which the interventions were based. More knowledge is needed regarding the mechanisms that promote sexual health, and explicitly describing the theoretical underpinning of studies is important in order to further the knowledge base of any research field (73). The theoretical frameworks or models that were mentioned in these sexual health promotion studies were: a social network intervention framework (peer-delivered intervention) (26, 40), game-based learning with a participatory design approach (41), Kelly's popular opinion leader model (30), peer education models (58), Pender's health promotion model (24), and a community engagement model (65).

### Most of the publications focused on general sexual health promotion, targeted youth, and used Facebook

The review also demonstrated that the majority of studies involving online social media for sexual health dealt with the topic in more of a general way or focused on increased STI testing. Only 15 of the publications dealt with the incurable STI, HIV, and seven dealt with curable STIs (chlamydia, syphilis, gonorrhea, or HPV). About 60% of the included publications specifically targeted young people (up to 29 years old), and these publications were mainly on STI health promotion in general. The studies that explicitly targeted adults were directed toward MSM/LGBT and mostly focused on HIV. We have found only a few intervention projects using social media for additional sexual health promotion topics, such as abortion and teenage pregnancy. These projects were carried out by NGOs. We believe that, in addition to health promotion on STIs, there especially is a need for further studies examining the effects of social media interventions for health promotion in other sexual health domains, such as maternal health, contraception, or female genital mutilation.

Although it is important to reach people at a young age to prevent early infection with STIs, this does not mean that other age groups should not be targeted. The problem of STIs primarily impacts young people and young adults, but it is also important to avoid STIs among adults older than 30. Although young people have been early adopters of online social media (74), today online social media are popular in all age groups, which means that online social media could be a good channel for sexual health promotion among older adults as well.

Studies from other health care fields utilizing social media have demonstrated positive effects on health outcomes dealing with wellness, obesity, and the management of chronic diseases (75). Thus, there is mounting evidence suggesting that online social media have a great potential in the field of health information and also with respect to some other types of health interventions. In the sexual health field, Facebook seems to be the most frequently used online social media channel, possibly because of the very high number of users (3). In this review, 44 papers (86%) involved Facebook as a channel for sexual health promotion—either as the only online social medium or in combination with other online social media. This review found that the use of Facebook is slightly higher than the 71% reported by Gold et al. (15) in a review of social media use for sexual health promotion published in 2011, when Facebook had 500 million active users worldwide (15).

Facebook and other social media are increasingly being used to reach specific populations for health promotion purposes. As evidence of the efficacy of this type of intervention for health promotion is increasing, organizations belonging to the public sector, NGOs, and other stakeholders might be encouraged to use social media more often for sexual health promotion and sexual health education.

Although the WHO reports that the majority of people suffering from STIs are located in Southeast Asia, sub-Saharan Africa, Latin America, and the Caribbean (1), the higher proportion (40) of the publications included in this review targeted populations from the US, Canada, Europe, Australia, and New Zealand. Only 10 of the publications reported on studies that had been carried out in other parts of the world, such as in Africa (Nigeria and Ghana) (60, 66, 67, 69), Southeast Asia (30, 41, 57), the Middle East (including Pakistan) (59, 61), and in South America (Peru) (43). One publication involved 79 countries (29). Most of the sexual health education interventions carried out in developing countries are organized by NGOs. There is a discrepancy as to where the largest number of people affected by STIs live, the worldwide distribution of social media users, and where the most studies are carried out. This may in part represent a question of resources that are available for sexual health promotion because about 55% of all worldwide social media users are located in Asia and the Middle East; 13% in Central and South America; and 6% in Africa; while only about a quarter of all social media users are distributed in Europe, North America, and Oceania (76). However, other issues, including social and cultural factors, are also relevant. Social and cultural factors are of importance regarding how sensitive topics are discussed in general (77, 78), and these factors might therefore influence the acceptability of using online social media for sexual health topics in different parts of the world.

### Limitations

Only 51 publications matched the inclusion criteria in this review. Although we believe that the search terms used allowed us to capture relevant publications in this field, we might have missed publications that were not identified with the search terms or that were not published in the journals or databases that were searched. Because we searched the websites of the NGOs manually, some projects might have been missed as might publications from NGOs whose websites were not specifically searched. Some sexual health promotion interventions and programs using social media were excluded from our review because they did not report any study outcomes.

The included publications had heterogeneous designs and purposes and reported different types of outcomes. The quality of the studies varied, and there were only four publications that reported on studies with a strong randomized design. However, these studies did report findings with important implications, including increased condom use and HIV testing. Due to the small number of studies included in this review that reported effects, and the differences among the included studies, it was not possible to compare effect sizes or to conduct a meta-analysis.

## Conclusions

Some studies have used online social media for sexual health promotion or STI prevention—either as a sole intervention or in combination with other interventions. Only a handful of studies have used a controlled randomization procedure. About a quarter of the publications identified promising results, and the evidence for the positive effect of social media interventions for promoting sexual health is increasing. However, there is a need for more studies with strong designs to increase the weight of the evidence and to move this emerging field forward.

## Supplementary Material

Use of social media for sexual health promotion: a scoping review

## Paper context

Social media are becoming increasingly important in sexual health promotion. This review shows that about a quarter of the studies reported effects, and the results relating to increased condom use and increased testing were the most convincing. However, few studies had RCT designs, and more strongly designed studies are needed to demonstrate the benefits of social media for sexual health promotion.
