# Supplementary material for: Use of social media for sexual health promotion: a scoping review
Source: Glob Health Action. 2016 Sep 19;9:10.3402/gha.v9.32193. doi: 10.3402/gha.v9.32193 (PMC5030258; doi:10.3402/gha.v9.32193)
Supplement: Use of social media for sexual health promotion: a scoping review [file GHA-9-32193-s001.docx]

**Appendix 1**

We developed a full electronic search strategy. We first searched 8 databases for studies published until the end of October 2015 using combination of search terms “sexual health promotion” or “sexual health education” with “social media”; “social networking”; “Facebook”; “Twitter”; “YouTube”; “Instagram”; and/or “Snapchat”. In table 1, we provide the search terms used, databases searched and a description of the results of the search.

**Table 1. Search strategy**

|  | **EMBASE** | **Pubmed*** | **PsychINFO** | **Applied Social Sciences Index and abstracts (ASSIA)** | **ProQuest Health & Medical Complete** | **British Nursing Index** | **Computer and information systems abstracts** | **MEDLINE (Ovid)** |
| --- | --- | --- | --- | --- | --- | --- | --- | --- |
| (Social media) AND (sexual health promotion OR sexual health education) | 351 | 276 | 215 | 109 | 76 | 8 | 4 | 4 |
| (Social networking) AND (sexual health promotion OR sexual health education) | 353 | 271 | 216 | 18 | 23 | 5 | 3 | 3 |
| (Facebook) AND (sexual health promotion OR sexual health education) | 353 | 271 | 216 | 10 | 11 | 3 | 3 | 3 |
| (Twitter) AND (sexual health promotion OR sexual health education) | 347 | 264 | 212 | 0 | 3 | 2 | 0 | 0 |
| (Youtube) AND (sexual health promotion OR sexual health education) | 345 | 261 | 211 | 2 | 0 | 0 | 1 | 0 |
| (Instagram) AND (sexual health promotion OR sexual health education) | 343 | 260 | 211 | 0 | 19 | 0 | 0 | 0 |
| (Snapchat) AND (sexual health promotion OR sexual health education) | 343 | 0 | 210 | 0 | 5 | 0 | 0 | 0 |

* Pubmed search was carried out for text word, and also for Mesh terms “Social Media”; “Social Networking”; and “Sex Education”

To capture grey literature, additional publications, conference proceedings and research reports were searched in additional databases:

- African Journals Online (AJOL). Search hits = 0
- COS Conference Papers Index. Search hits = 4
- Directory of Open Access Journals (DOAJ). Search hits = 17
- Clinicaltrials.gov. Search hits = 272
- Conference proceedings published in the journals Sexually Transmitted Diseases; Sexually Transmitted Infections; and International Journal of STD & AIDS:
  - 4^th^ Joint BASHH-ASTDA meeting, 2012 (218 abstracts)
  - BASHH 2013 (194 abstracts)
  - STI & HIV World Congress 2013 (1216 abstracts)
  - 2014 STD Prevention Conference (533 abstracts)
  - Infection Prevention 2014 (125 abstracts)
  - BASHH 2015 (542 abstracts)
  - STI & HIV World Congress 2015 (296 abstracts)
  - 29^th^ European Conference on Sexually Transmitted Infections, 2015 (190 abstracts)
- Programmes evaluation reports referring to the use of social media for sexual health promotion or sexual health education, were manually searched in websites of the following non-governmental organizations (NGOs):
  - International Planned Parenthood Federation (IPPF)
  - Population Council
  - World Health Organization (WHO)
  - United Nations Population Fund (UNFPA)
  - Youth Leading the HIV & Hep C Movement (YouthCO)
  - FHI360
  - The Initiative for Equal Rights (TIER)
